# Surface Property Modification of Silver Nanoparticles with Dopamine-Functionalized Poly(pentafluorostyrene) via RAFT Polymerization

**DOI:** 10.3390/polym8030081

**Published:** 2016-03-14

**Authors:** Ka Wai Fan, Anthony Michael Granville

**Affiliations:** Centre for Advanced Macromolecular Design, The School of Chemical Engineering, University of New South Wales, Kensington NSW 2052, Australia; k.w.fan@unsw.edu.au

**Keywords:** RAFT polymerization, poly(pentafluorostyrene), silver nanoparticles, glycopolymer

## Abstract

This research aims to synthesize a dopamine-functionalized macromolecular anchor to perform surface modification on the target nanostructures. A molecular anchor, 3,4-dichloro-1-[2-(3,4-dihydroxyphenyl)ethyl]-*1H*-pyrrole-2,5-dione, was successfully synthesized from dopamine and 2,3-dichloromaleic anhydride. The anchor acted as a linkage to couple the chains of poly(pentafluorostyrene) (PPFS) which were synthesized via reversible addition fragmentation chain transfer (RAFT) polymerization. Modification was successfully performed to silver nanoparticles (AgNPs) by deposition of the dopamine-functionalized coupled PPFS onto the surface of the particles. The modified AgNPs had demonstrated improved dispersibility in organic solvent due to the hydrophobic nature of PPFS. To modify the surface chemistry of the nanoparticles further, thioglucose was grafted onto the structure of the coupled PPFS via thiol-fluoro nucleophilic substitution at the para-position of the pentafluorophenyl groups on the monomer units. The presence of sugar moieties on the coupled PPFS increased its hydrophilicity, which allowed the modified AgNPs to be readily dispersed in aqueous solvent.

## 1. Introduction

Nanoparticles are clusters of atoms with a size range of 1–100 nm [1], and exhibit unique physical and chemical properties that would not be revealed in the bulk material [2,3,4]. Metal nanoparticles (MNPs), in particular, have aroused a vast interest in exploring their use for various applications, with research on silver nanoparticles (AgNPs) growing in popularity. For instance, AgNPs have a much higher negative reduction potential than bulk silver. Such a property, along with the high surface area to volume ratio, allows them to be used as the active catalyst to reduce other chemical species such as dyes and aromatic nitro compounds [2,3]. In terms of biomolecule sensing, AgNPs have been used as nanoprobes for determining the concentration of dopamine [4,5,6]. Such technology is selective towards dopamine because of the strong AgNP-catechol interaction and more efficient than other traditional techniques based on spectroscopy and HPLC instrumentation. A more sensitive measurement can also be achieved due to the higher extinction coefficients of AgNPs compared to other nanoparticles, e.g., gold, of the same size [5], which is preferable for colorimetric analysis [4,5].

Silver and its salts have been well known for their broad spectrum of antibacterial ability [7,8,9,10,11,12]. Silver nitrate, for example, has been used as an active ingredient in wound dressings and burn ointments [11]. Medical devices have been incorporated with silver-containing materials to prevent biofilm formation, thus reducing fouling and inflammation in the body [13]. Recently, the surge of antibiotic-resistant bacterial strains has aroused public health concern. There is a growing interest within the biomedical field in using silver (in the form of AgNPs) as a more effective antibacterial agent than antibiotics [9,11]. The large surface area to volume ratio of AgNPs promotes interaction between the nanoparticles and the constituents of the outer membrane of bacterial cells, which enhance their antibacterial efficacy [8,14]. As AgNPs penetrate through the cell, silver ions (Ag^+^) are released to enhance the antibacterial activity [11,14]. Bacteria death can occur when: (1) the structural disruption of the cytoplasm at which DNA (deoxyribonucleic acid) is condensed and loses replication ability [9,15]; and (2) the deactivation of proteins and enzymes by the binding of Ag^+^ to the thiol groups on the biomolecules to interfere with normal metabolic activities, such as the synthesis of adenosine triphosphate (ATP) [7,16].

AgNPs, however, are seldom applied directly for a number of reasons. Firstly, hydrophobic AgNPs tend to aggregate even if they are dispersed in solution due to the high surface energy and van der Waals forces unless a stabilizer has been applied [2]. Aggregation alters the particle size and hence reduces the available surface area. Since the release rate of Ag^+^ by AgNPs is highly dependent on particle size, broadly dispersed AgNP aggregates would lead to incomplete control of the release of Ag^+^ in the medium and affect the antimicrobial efficacy [13,14,15]. Furthermore, silver is cytotoxic at high concentrations (>30 ppm). Cell viability and mitochondrial activity might be decreased as a result of exposure to AgNPs due to the combined effect of increased production of reactive oxygen species, depletion of the glutathione, and lipid peroxidation [16].

Immobilization of AgNPs on a physical surface can minimize the potential cytotoxic effect due to exposure [16]. Immobilization also ensures high availability and uniform distribution of particle surface area by preventing AgNP aggregation, which permits better control of Ag^+^ release [14,15]. The most common anchoring agents for AgNP immobilization are surfactants and polymers [7,16]. Jain and Pradeep attempted to immobilize AgNPs onto poly(urethane) foams in preparation of an antibacterial water filtration membrane [17]. The membrane was able to purify water to the quality that is in line with the World Health Organization requirements for drinking water. Polymeric composites such as polyelectrolytes and block copolymers have also been used for the immobilization of AgNPs [14,15]. Misra *et al.* attempted to prepare an antibacterial composite by immobilizing AgNPs on poly(ethylene)-*block*-poly(ethylene oxide) that has been deposited on carbon nanotubes. The prepared composite has demonstrated a four-magnitude increase in antibacterial activity compared to the *ex situ* precipitated bare AgNPs [15].

The immobilization of AgNPs can also suffer from the intrinsically poor metal/substrate adhesion resulting in the nanoparticle gradually being displaced from the substrate [13]. There have been novel approaches to act on such limitations by incorporating adhesive functional groups into the structure of the substrate. Dopamine has become the ligand of interest for such purposes since it has previously demonstrated strong interactions with AgNPs [8,11,13]. Being a transition metal, AgNPs form coordination complexes with various ligands, e.g., the citrate ions which act as stabilizers to the synthesized nanoparticles [15,18]. The catechol group on dopamine behaves as a bidentate binder to the surface of AgNPs [5]. Dopamine also exhibits versatile chemistry, whereby the primary amine functional group allows for further modification of the dopamine molecule, and hence the occupied surface [6,19]. The dopamine-modified surface can thus provide active sites where further functionalization can take place. In biomedical applications, substrates are often grafted with polymer chains to improve their biorecognition. Glycopolymers, which consist of a synthetic polymeric backbone and pendant sugar moieties, have drawn attention from various researchers for this particular purpose [20]. The pendant carbohydrate units behave as ligands to a range of protein receptors, thereby generating unique biochemical properties for these polymers.

Being able to functionalize and modify the surface properties the surface of AgNPs would be beneficial to further explore their potential applications. As such, research had been carried out to verify the potential use of a macromolecular anchor as the substrate, which contains dopamine in its structure, for AgNPs and the subsequent modification of the nanoparticle surface. In this work, dopamine was reacted with 2,3-dichloromaleic anhydride to synthesize 1H-pyrrole-2,5-dione-3,4-dichloro-1-[2-(3,4-dihydroxyphenyl)ethyl]- as a molecular anchor (dopamine-dichloromaleic anhydride, DA-DCMA anchor). 2,3,4,5,6-Pentafluorostyrene (PFS) was chosen as the monomer to synthesize the polymeric backbone, poly(pentafluorostyrene) (PPFS), *via* reversible addition fragmentation chain transfer (RAFT) polymerization. Thiol-chloro nucleophilic substitution was performed to couple the synthesized PPFS by using the DA-DCMA anchor as a linkage. The coupled PPFS was equipped with the chelating ability of dopamine and anchored onto dispersed AgNPs to verify whether the modified nanoparticles would disperse in organic solvent. A portion of the coupled PPFS was functionalized with sugar moieties by reacting thioglucose at the para-position of the pentafluorophenyl group of the monomer units *via* thiol-fluoro nucleophilic substitution [21]. The resulting glycosylated PPFS (GPPFS) was then anchored onto the AgNPs. The pendant glucose units increased the hydrophilicity of the PPFS backbone, which allowed the GPPFS-modified nanoparticles to be dispersed in aqueous based solvents. It is envisioned that these aqueous dispersible AgNPs would be capable of enhanced biomedical and environmental applications due to their low aggregation properties.

## 2. Materials and Methods

### 2.1. Materials

Dopamine hydrochloride (99%, DA), 2,3-dichloromaleic anhydride (97%), 1-hexylamine (99%), sodium citrate tribasic (≥99%), and fluorobenzene were purchased from Sigma-Aldrich (Sydney, Australia) and used as received without any further purification. Silver nitrate and triethylamine (TEA) were purchased from Univar (Sydney, Australia) and also used as received. 2,2′-azobis(isobutyronitrile) (AIBN, Sigma-Aldrich, 97%) was purified by recrystallization from cold methanol prior to use. Pentafluorostyrene (PFS, Oakwood Products, 99%) was passed through a column of activated basic alumina prior to use to remove inhibitor. 4-cyanopentanoic acid dithiobenzoate (CPADB) was synthesized as described in the literature [22]. 1-thio-β-ᴅ-glucose sodium salt (GluSNa) was prepared from 1-thio-β-ᴅ-glucose tetraacetate (AcO-GluSH) according to literature method [23]. All solvents were laboratory reagent grade and used as received without any further purification.

### 2.2. Synthesis of AgNPs

The AgNPs used in the research were synthesized *via* the MCR method [4]. A solution which contained silver nitrate (1 mL, 1 wt %) in 100 mL of distilled water was heated to 130 °C under vigorous stirring. Once the solution mixture began to boil, a sodium citrate aliquot (6 mL, 1 wt %) was added rapidly to the solution. A gradual color shift, after citrate addition, from colorless to murky grey-yellow was observed. The reaction mixture was boiled for another 10 min, and then slowly cooled to room temperature. The synthesized AgNPs were left dispersed in the solution and stored in the dark.

### 2.3. Synthesis of DA-DCMA Anchor

The DA-DCMA anchor was synthesized by modifying the method described by Jones *et al.* [24]. Dopamine hydrochloride (1.00 g, 5.27 mmol) was added to a solution which contained 2,3-dichloromaleic anhydride (1.00 g, 5.99 mmol) dissolved in 50 mL of anhydrous acetic acid. The reaction mixture was refluxed at 120 °C for 24 h under vigorous stirring. The color of the mixture changed from a brownish-yellow to clear dark green as the reaction progressed. Acetic acid was partially removed by blowing air onto the resulting solution to obtain a dark green slurry, which was washed with toluene and filtered to generate a yellowish-orange filtrate. A pale yellow solid of the DA-DCMA anchor remained after the removal of toluene from the filtrate. The solid was recrystallized from a solution mixture of distilled water and methanol. Golden yellow crystals were obtained after filtration. The crystals were washed with distilled water and dried under vacuum overnight.

### 2.4. RAFT Polymerization of PFS and Subsequent Aminolysis of RAFT End Groups

For the polymerization, AIBN (10.6 mg, 0.06 mmol) and CPADB (90.1 mg, 0.32 mmol) were added to a solution which contained PFS (2.1 mL, 2.95 g, 15.21 mmol) in 13 mL of fluorobenzene. The reaction mixture was chilled while being degassed by bubbling nitrogen gas through the solution for 20 min. The polymerization was then carried out by heating the mixture at 70 °C for 24 h. The reaction was quenched, and the solvent was removed under vacuum. The obtained solid polymer was analyzed by gravimetric as well as Gel Permeation Chromatography (GPC) analysis to determine molecular weight and monomer conversion.

Aminolysis was performed on the RAFT generated PPFS to reduce the dithiobenzoate end-group to a thiol [25]. 30 µL of 1-hexylamine (0.22 mmol) was added to a reddish pink solution containing the PPFS (0.60 g, *M*_n_ ≈ 2800 g·mol^−1^, 0.21 mmol) dissolved in 20 mL of THF. The color of the solution progressively changed from pink to yellow as aminolysis reaction proceeded. The reaction was performed for 6 h at room temperature to allow for complete reduction of the RAFT end groups.

### 2.5. Coupling PPFS Chains to DA-DCMA Anchor and Glucosylation

The coupling of PPFS was performed under similar conditions to those described by Jones *et al.* [24]. The DA-DCMA anchor (40 mg, 0.17 mmol) was added to the yellow solution of thiol end group PPFS. The reaction was carried out overnight and at 40 °C to drive the chain coupling to completion. A yellow solid remained after the solvent was removed, which was subsequently rinsed with small portions of methanol, followed by distilled water. The dried, pale-yellow solid was analyzed by GPC and ^1^H NMR to confirm the coupled product was obtained.

The GluSNa (0.28 g, 1.28 mmol) was added to a solution containing the coupled PPFS (0.20 g, *M*_n_ ≈ 4500 g·mol^−1^, 0.07 mmol) dissolved in 10 mL of DMF. To this solution, 0.45 mL of TEA (3.2 mmol) was added to the resulting mixture, which was then heated at 40 °C for 8 h. The solvent was removed under vacuum and the subsequent solids of glycosylated PPFS (GPPFS) were retained for GPC and NMR analysis.

### 2.6. Anchoring of Coupled Polymer Chains to AgNPs

AgNPs were anchored with both coupled PPFS chains as well as coupled GPPFS chains, to determine any differences in grafting density and solubility. For the PPFS anchoring, 60 mL of the AgNPs aqueous dispersion was added to a solution of THF (10 mL) containing the coupled PPFS (50 mg). The reaction was carried out at room temperature for 18 h under vigorous stirring. THF was removed from the solution by rotary evaporation. The aqueous solution was then centrifuged, concentrated by removing the supernatant, and washed with 10 mL of methanol. The washing procedure was repeated using 10 mL of distilled water. The solution was freeze-dried to obtain the modified AgNPs (PPFS@AgNPs).

A similar procedure was performed for the glycosylated PPFS, where 50 mg of GPPFS was dissolved in 70 mL of the AgNP aqueous dispersion. The anchoring reaction was carried out at 40 °C for 18 h under vigorous stirring. The modified AgNP solution (GPPFS@AgNPs) was purified and freeze-dried under similar conditions to the PPFS anchoring procedure. All anchored AgNPs were characterized using DLS, ATR-FTIR, and TGA techniques.

### 2.7. Characterization

Attenuated Total Reflectance Fourier Transform Infrared (ATR-FTIR) spectroscopy was performed on a Bruker IFS 66/S single-beam spectrometer (Kensington, Australia) using diffuse reflectance sampling accessories. Spectra were recorded at a resolution of 4 cm^−1^ and 32 scans were collected per sample.

Dynamic Light Scattering (DLS) spectrometry was performed with a Malvern Zetasizer Nano ZS (Kensington, Australia). The device was equipped with a 4mW He–Ne laser (λ = 632.8 nm) and calibrated using titanium oxide as the reference material (RI = 2.400, absorption = 0.01). Three measurements were performed for each sample, and each measurement consisted of 12 scans. The refractive index (RI) of the samples was set at 1.59 for AgNPs with a material absorption of 0.01.

Gel Permeation Chromatography (GPC) was performed on two separate Shimadzu modular systems (Kensington, Australia), one utilizing THF [HPLC grade, 250 ppm 2,6-dibutyl-4-methylphenol (BHT)] as the eluent and the other utilizing DMAc [HPLC grade, 0.03% *w*/*v* LiBr, 0.05 wt %. BHT]. The THF GPC was equipped with a CTO-10AC column oven (Kensington, Australia) operating at 40 °C and four Phenogel columns (102, 103, 104, 106 Å pore size). The DMAc GPC was equipped with a CTO-10A column oven (Kensington, Australia) operating at 50 °C and four PL (Styragel) columns (500, 103, 104, 105 Å pore size). Both systems were operated at a flow rate of 1 mL·min^−1^ equipped with a Polymer Laboratories guard column (5 µm bead size, 50 × 7.8 mm) and a RID-10A refractive index detector (Kensington, Australia). The systems were calibrated against commercially available polystyrene standards (0.5–1000 kDa) purchased from Polymer Laboratories (Hopkins, MN, USA). The samples were prepared at a concentration of 2–3 mg·mL^−1^ and filtered through a 0.45 µm filter prior to injection.

^1^H, ^13^C and ^19^F Nuclear Magnetic Resonance (NMR) spectroscopy data of the synthesized samples were generated by a Bruker DPX-300 spectrometer (Kensington, Australia) at a resonance of 300 MHz at 25 °C. Different deuterated solvents purchased from Cambridge Isotope Laboratories, Inc. (Tewksbury, MA, USA), have been used to prepare the NMR samples according to the nature of the sample being tested.

Scanning Electron Microscopy (SEM) images of the AgNPs were acquired with a Hitachi S3400 electron microscope (Kensington, Australia) with a chromium surface coating.

Thermogravimetric Analysis (TGA) was performed with a PerkinElmer Simultaneous Thermal Analyzer (STA) 6000 (Kensington, Australia). The instrument heat profile used was a heating step from 30 to 100 °C at 5 °C·min^−1^ followed by heating to 900 °C at 10 °C·min^−1^ for thermal degradation of the sample. A control was run for each nanocomposite using the unmodified sample.

## 3. Results and Discussion

The nanoparticles synthesized via the MCR method were relatively large in size, which gave a murky appearance to the AgNP solutions. The solution was dispersed in distilled water for all DLS measurements and exhibited unimodal size distributions upon replicate measurements. Some degree of batch variation was observable from the particle size measurements, where the Z-average diameter varied from 22 to 49 nm (Table S1). The relatively large size of the synthesized AgNPs, however, had the advantage of providing a larger surface area for modification. In addition to particle size analysis, the AgNPS were characterized by ATR-FTIR spectroscopy (Figure S1). While silver may not respond well to infrared radiation, residual stabilizers and reagents from the particle synthesis would. For instance, the strong absorption peak due to OH stretch at 3600–2500 cm^−1^ is likely due to the presence of citric acid stabilizer. This is in addition to the cluster of peaks at 1300–1000 cm^−1^, indicative of C–O stretch also due to citric acid. Finally, the peaks at 1550 and 1370 cm^−1^ were attributed to residual nitrate ions since the sample had not been purified.

The synthesis of the DA-DCMA anchor was adopted from a similar reaction scheme reported by Jones *et al.* [24]. The yellow solid of the DA-DCMA anchor was extracted into toluene from the slurry by filtration, and recrystallized from a 50:50 mixture of methanol and distilled water resulting in a product yield of 54.7%. Characterization of the anchor was performed using ^1^H NMR (Figure S2), ^13^C NMR (Figure S3) and ATR-FTIR (Figure S5), and a summary of the peak analysis can be found in the Supplementary Materials (Tables S2 and S3). As illustrated by the NMR spectrum of dopamine (Figure S4), the peak position of the C5 protons shifted from 2.92 to 3.62 ppm after the anchor synthesis reaction was complete, due to an increase in the electronegativity around the protons. The shifted peak, as well as all NMR and ATR-FTIR analysis, proved that the anchor was successfully synthesized at the terminal amino group of dopamine.

**Scheme 1:**
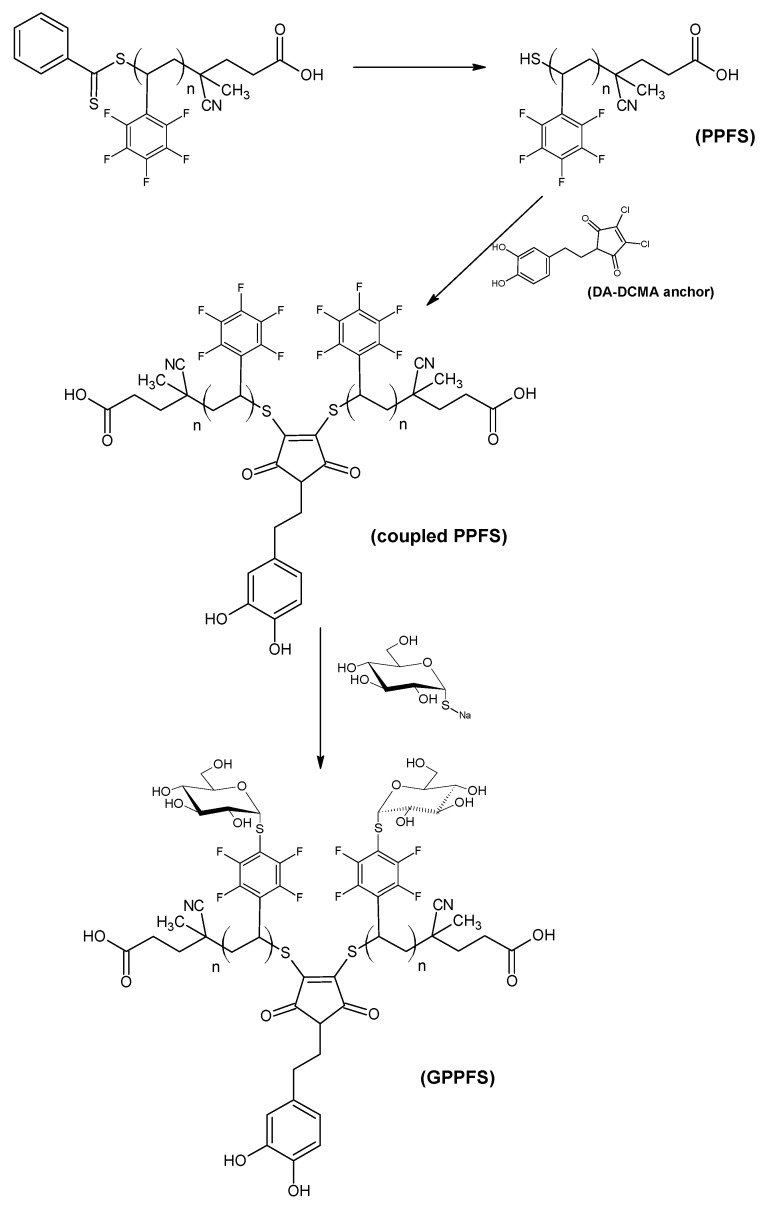
Reaction steps for generating poly(pentafluorostyrene) (PPFS) y-brush with dopamine based anchor.

**Figure 1:**
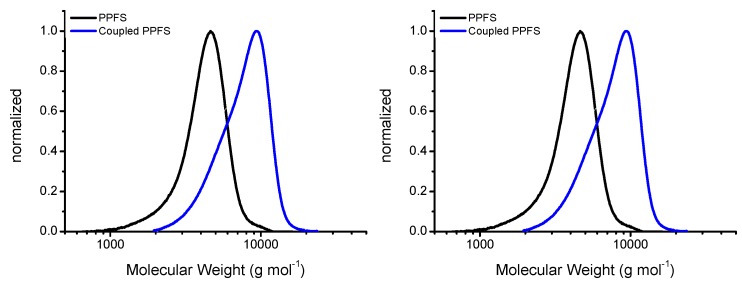
Gel Permeation Chromatography (GPC) analysis of PPFS chains (black) and coupled PPFS (blue) with DA-DCMA anchor.

The prepared CPADB RAFT agent was used in the synthesis of PPFS. These polymerization reactions yielded 1.15 g of dried PPFS, corresponding to an overall conversion of 46.3%. The polymer was characterized by ^1^H NMR spectrometry (Figure S6 and Table S4). There were 13 monomer units within the PPFS chain, based on this analysis, which corresponds to a molecular weight of approximately 2800 g·mol^−1^. A similar molecular weight (*M*_n_ = 3080 g·mol^−1^, *Ð* = 1.13) was also obtained from the GPC analysis which shows a unimodal distribution (Figure 1). Upon aminolysis of the RAFT end group, these monomodal PPFS chains were used to couple to the DA-DCMA anchor as shown in the reaction scheme (Scheme 1). As the aminolysis reaction commenced, the solution color shifted from pink, indicative of the CPADB RAFT agent and polymers, to pale yellow. The change in color was due to the reduction of the dithiobenzoate RAFT agent end-group to a thiol group, gaining the characteristic yellow color of thiolated compounds.

The coupling step was carried out in the same reaction vessel since the DA-DCMA anchor is soluble in THF. The procedure was adopted from Jones *et al.* [24] in which 2,3-dibromomaleimide was used as the linkage with the thiol groups. Nucleophilic substitution of organic bromide with thiol proceeds more readily than that of organic chloride since the bromine atom is a better leaving group. Thus, the reaction mixture was heated to 40 °C overnight to drive the coupling to completion. An increase in intensity of the yellow color was observable after the coupling reaction. The coupled PPFS was analyzed using GPC and the results were compared to the original RAFT generated PPFS chains (Figure 1). The GPC results showed a slightly greater than doubling in the molecular weight (*M*_n_ = 6450 g·mol^−1^) as well as a broadening, and tailing, of the GPC trace resulting in a *Ð* of 1.22 (Table S5). The increase in dispersity and tailing is assumed to be due to a less than 100% coupling reaction. While the PPFS chains are relatively low molecular weight, it is possible that the coupling of one chain to the DA-DCMA anchor could sterically hinder the coupling of the second chain to the anchor. Unfortunately, even when the reaction time was increased to 36 h, no appreciable decrease in the tailing of the GPC trace was observed. Thus, it was determined that a majority of the chains were coupled, and binding of the anchors to the AgNPs was investigated. Further confirmation of the coupling was provided by the ^1^H NMR analysis of the sample, showing an almost exact doubling in protons on the polymer backbone (Figure S7 and Table S6).

**Figure 2:**
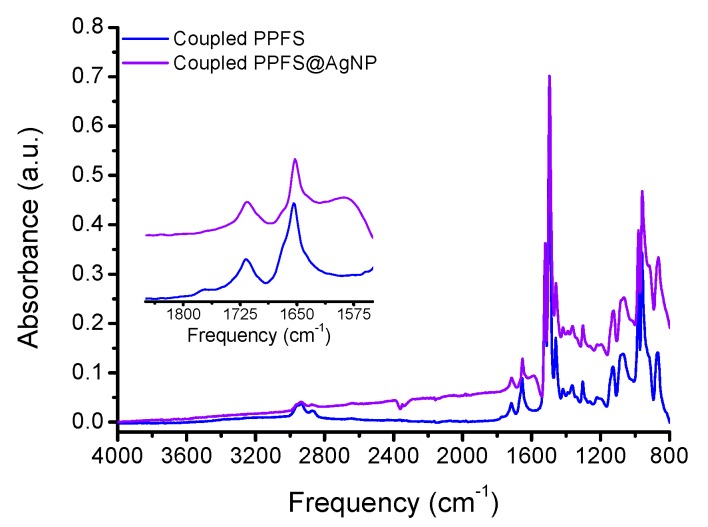
Attenuated Total Reflectance Fourier Transform Infrared (ATR-FTIR) analysis of coupled PPFS and anchored PPFS@AgNPs.

**Figure 3:**
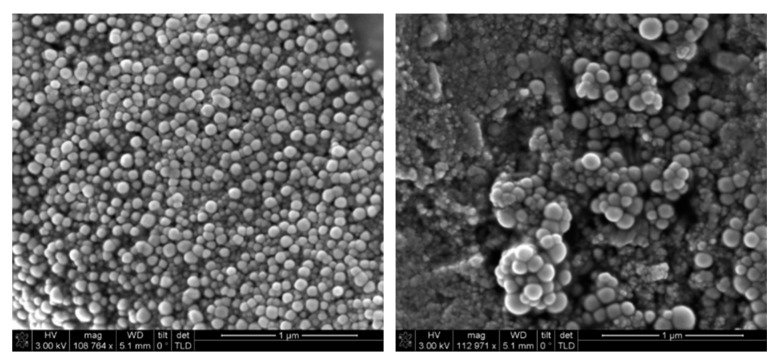
SEM image of AgNPs (**left**) and PPFS coupled AgNPs (PPFS@AgNPs) (**right**).

The AgNP aqueous aliquot mixed well with the coupled PPFS THF solution, and vigorous stirring was also applied to enhance the particle dispersion and increase grafting density. The surface grafting of the grey AgNP solution was performed for 18 h and the particles were analyzed ATR-FTIR, DLS, and TGA to determine the extent of grafting. The ATR-FTIR spectrum of the PPFS@AgNPs clearly shows features of the PPFS chains (Figure S8) and the loss of the residual citric acid and nitrate ions from the bare AgNP synthesis (Figure 2). In addition to the FTIR analysis, the particle size was analyzed in both water and THF, based on the solubility of the PPFS chains (Figure S9). When DLS measurements were performed in water, a large amount of particle agglomeration occurred resulting in particles sizes in excess of 900 nm in diameter (Table S7). However, when THF was used, the agglomeration was visibly reduced, and an average particle size of 140 nm was observed. While this is considerably higher than the 22–49 nm diameter bare AgNPs generated, as can be seen in the SEM images (Figure 3), some aggregation is still noticeable in the samples.

**Figure 4:**
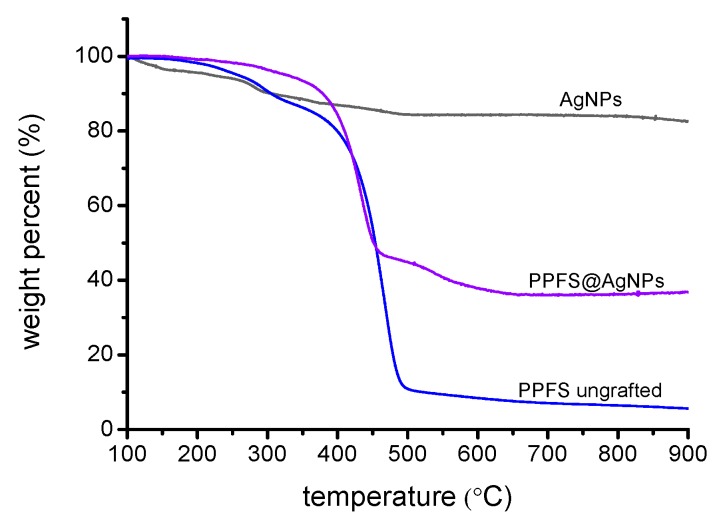
TGA analysis of bare AgNP, RAFT generated DA-DCMA coupled PPFS chains, and PPFS coupled to AgNPs (PPFS@AgNPs).

To confirm the grafting density and thermal stability of the PPFS@AgNPs, TGA analysis was performed. As can be seen in Figure 4, a relatively small mass loss is observed for the bare AgNPs which corroborates the FTIR data suggesting that residual citric acid and nitrate groups remain from the particle synthesis. The DA-DCMA coupled PPFS chains, prior to grafting to the AgNPs, exhibit two distinct mass losses, a shallow slope occurs at 238 °C possibly due to the breaking of the sulfide-linkage between the PPFS chains and the DA-DCMA anchor. The steep slope occurring at 408 °C would be due to the degradation of the backbone of PPFS. Finally, when the PPFS@AgNPs were analyzed, a single major mass loss slope is observed, occurring at roughly 379 °C, with a similar gradient as the DA-DCMA coupled PPFS degradation. This would suggest that this major mass loss from the AgNP surface is an amalgamation of the degrafting of the PPFS anchors as well as the decomposition of the PPFS backbone. The total mass loss for the PPFS@AgNPs, attributed to the organics on the particle surface, equates to 52%. Thus, it was calculated that the total grafting density of the PPFS chains on the AgNP surface to be 22 chains·nm^−2^ (Table S8).

While the grafting of PPFS chains to AgNPs improved the disperability of these particles in organic solvents, in order to make these particles useful for medical purposes, their dispersability in aqueous media needs to be addressed. Thus, the glucosylation of the PPFS chains was investigated using the aromatic substitution of the para-fluorine group with thiol compounds. As reported by Becer *et al.* [26], the glycosylation of PPFS can occur readily at room temperature. However, the sodium thioglucose (GluSNa) reagent has a low solubility in DMF, the solvent for the reaction. Therefore, the reaction mixture was heated to 40 °C for the duration of glycosylation to ensure a high conversion. As the DA-DCMA coupled PPFS chains were dissolved in DMF and reacted with GluSNa, the pale yellow color of the mixture was observed to turn dark brown with some undissolved GluSNa suspended. DMAc GPC and ^1^H NMR analysis have been performed on the isolated brown crystalline product of coupled glucosylated PPFS (GPPFS).

According to the GPC analysis, the molecular weight of the GPPFS had increased significantly after glycosylation (Figure S10). The measured value (*M*_n_ = 86,500 g·mol^−1^; *Ð* = 1.66) was significantly higher than the estimated value (*M*_n_ = 11,600 g·mol^−1^; *Ð* = 1.23) based on full conversion of all the repeat units in the coupled chain. While it is possible that the individual chains vary greatly in their degree of conversion, the variation in the molecular weight is likely due to the differences in the radius of gyration for the GPPFS compared to the styrene calibration standard. As such, the GPC unit may not be able to accurately determine the molecular weight for GPPFS. Thus, a more accurate determination of the resulting molecular weight after glucosylation could be found through NMR analysis of the polymer chains. ^1^H NMR (Figure S12) and ^19^F NMR (Figure S13) analyses were performed on the isolated brown crystalline product from glycosylation of the coupled PPFS to investigate this. Peaks belong to the GPPFS are identified as followed (in δ ppm): CH and CH2 (backbone; 2.05 (72H); combined broad signal). The proton ratio between CH and CH2 group on the backbone is 1:2, so there are 24 protons which were originated from the CH group (*i.e.*, 24 monomer units in the polymer chain). The result agreed with the integration shown in the ^1^H NMR spectrum of the coupled PPFS (Figure S7). Furthermore, the weak signals at 4.50–6.00 ppm were possibly due to the protons on the sugar moieties [26]. Therefore, the prepared GPPFS should exist in the isolated brown crystal. According to the NMR results, the estimated molecular weight of the GPPFS is 9820 g·mol^−1^ (based on deconvolution of the ^19^F NMR peaks [26] and yielding roughly 70% conversion of the PFS units to thiosugar units, Figure S13), which is in good agreement with the estimated molecular weight based on the PPFS starting material. After the successful glucosylation of the coupled PPFS chains (Figure S11), the GPPFS chains were anchored to the AgNPs in a similar fashion as the PPFS chains. The now slightly hydrophilic chains were anchored to the surface and analyzed using ATR-FTIR, DLS, and TGA analysis in addition to observing the disperability of the particles in aqueous media.

**Figure 5:**
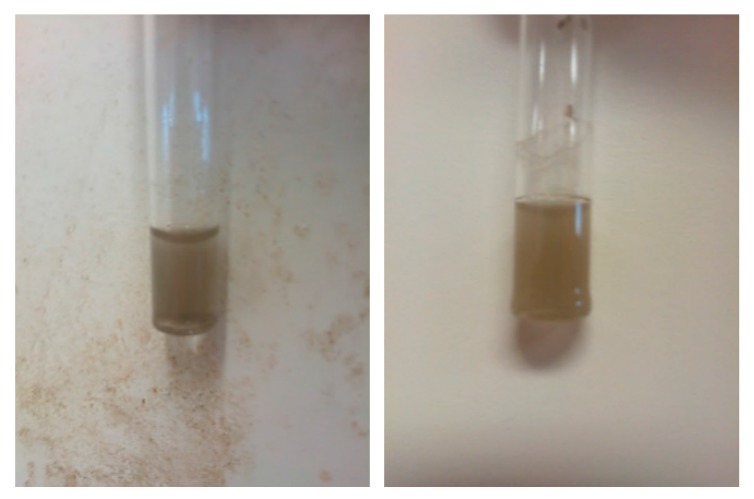
Dispersion of modified GPPFS@AgNPs in water before (**left**) and after (**right**) sonication.

The ATR-FTIR spectrum of GPPFS-AgNPs (Figure S14) highly resembles that of PPFS-AgNPs. The absorption peaks for the aromatic C–C stretch around 1480 cm^−1^ and the backbone CH stretch at 3000–2800 cm^−1^ of PPFS were observable. Furthermore, there was a noticable increase in the OH stretch absorption at 3600–3000 cm^−1^, a clear indicator that GPPFS was successfully anchored to the AgNP surface. Since GPPFS would be more hydrophilic than the coupled PPFS due to the presence of sugar moieties, distilled water was used as the dispersant to prepare sample for DLS analysis of the GPPFS@AgNPs. Although the DLS measurements showed a high polydispersity (0.66) and a rather large diameter (600 nm) when compared to the PPFS@AgNP samples, the particles are easily dispersed in the aqueous media requiring only shaking, rather than vigorous sonication, to disperse the samples. This is evident from the photos of the solutions in Figure 5, showing no particles aggregating or precipitating from the solution as was typical for the PPFS@AgNP samples.

**Figure 6:**
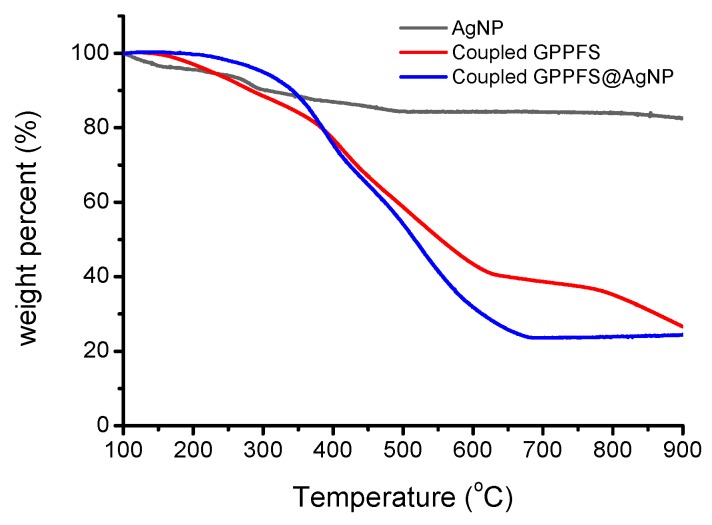
TGA analysis of bare AgNP, DA-DCMA coupled glycosylated PPFS (GPPFS) chains, and GPPFS coupled to AgNPs (GPPFS@AgNPs).

In addition to the aqueous dispersability of the modified AgNPs, the thermal stability of the glucose modified particles was investigated. As seen in Figure 6, the thermal degradation of the prepared GPPFS began at about 350 °C and continued to 600 °C and is the main weight loss for the polymer. When compared to the GPPFS@AgNPs, this major mass loss and onset degradation for the glucosylated polymer chains is virtually the same when anchored to the AgNP surface. However, when the mass loss for the GPPFS@AgNPs is compared to the PPFS@AgNPs samples, roughly 76% mass loss due to organics is observed compared to 52% for PPFS@AgNPs. While the GPPFS is significantly larger in both molecular weight and radius of gyration when compared to PPFS, the difference relates more heavily to a difference in grafting density for the two samples (Table S9). When the grafting density is calculated, as done for the PPFS@AgNPs samples, it is found that the glucosylated chains have close to half the grafting density (12 chains·nm^−2^). Thus, while the glucosylation of the PPFS chains enable for improved water dispersability of the AgNPs, the glucosylation results in a reduction in the grafting density due to increased steric hinderance of the chains. This reduced grafting density could affect nanoparticle uptake and cell interaction, although this is an aspect for current ongoing research for these materials.

## 4. Conclusions

We have successfully synthesized a dopamine based anchor capable of easily coupling to RAFT generated polymer chains through thiol coupling reactions. The anchor was reacted to PPFS chains which were prepared via RAFT polymerization and have been treated with aminolysis. The resultant coupled polymer exhibited an increase in molecular weight, which was determined by GPC and ^1^H NMR analyses. The successful surface modification of AgNP substrates has been achieved as proved by results obtained from ATR-FTIR, DLS and TGA analyses. In particular, the DLS results obtained for PPFS-AgNPs indicated that the polymer bound onto the surface allowed the modified-AgNPs to be dispersed more readily in organic solvent (THF in this case), in which non-modified-AgNPs would normally form aggregates. Therefore, surface modification of nanoparticles to improve the solubility (or interaction with the intended solvent) is achievable.

To increase their dispersion in aqueous media, a glycopolymer from the coupled PPFS was generated to increase the hydrophilicity. The ^19^F NMR and GPC (in DMAc) results suggested that the product obtained was glycosylated PPFS. While anchoring of the GPPFS to the AgNPs was successful and resulted in the desired increase to aqueous dispersion of the material, a significant reduction in the grafting density was observed. This decrease grafting density is the area of further investigations to determine its impact on other aspects of the nanoparticle system.

## Supplementary Materials

Supplementary materials can be found at www.mdpi.com/2073-4360/8/3/81/s1.
