# Supplementary material for: Surface Property Modification of Silver Nanoparticles with Dopamine-Functionalized Poly(pentafluorostyrene) via RAFT Polymerization
Source: Polymers (Basel). 2016 Mar 14;8(3):81. doi: 10.3390/polym8030081 (PMC6432553; doi:10.3390/polym8030081)
Supplement: Supplementary file 1 [file polymers-08-00081-s001.pdf]

Supplementary Materials: Surface Property Modification of Silver Nanoparticles with Dopamine-Functionalized Poly(pentafluorostyrene) via RAFT Polymerization

Ka Wai Fan and Anthony Michael Granville

Table S1. DLS measurement of AgNPs synthesized via MCR.

Sample name	Z-Average size (d. nm)	PDI	Peak (d. nm)	% Number
AgNPs (MCR)—3rd Batch	49	0.424	10	100.0
AgNPs (MCR)—2nd Batch	38	0.462	10	100.0
AgNPs (MCR)—1st Batch	22	0.550	5	100.0

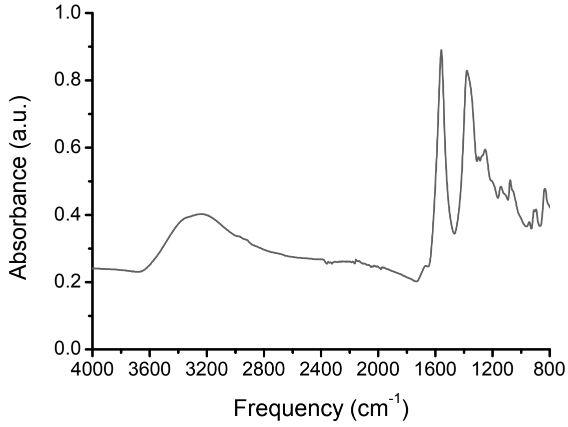

Figure S1. ATR-FTIR spectrum of AgNPs synthesized via the MCT method.

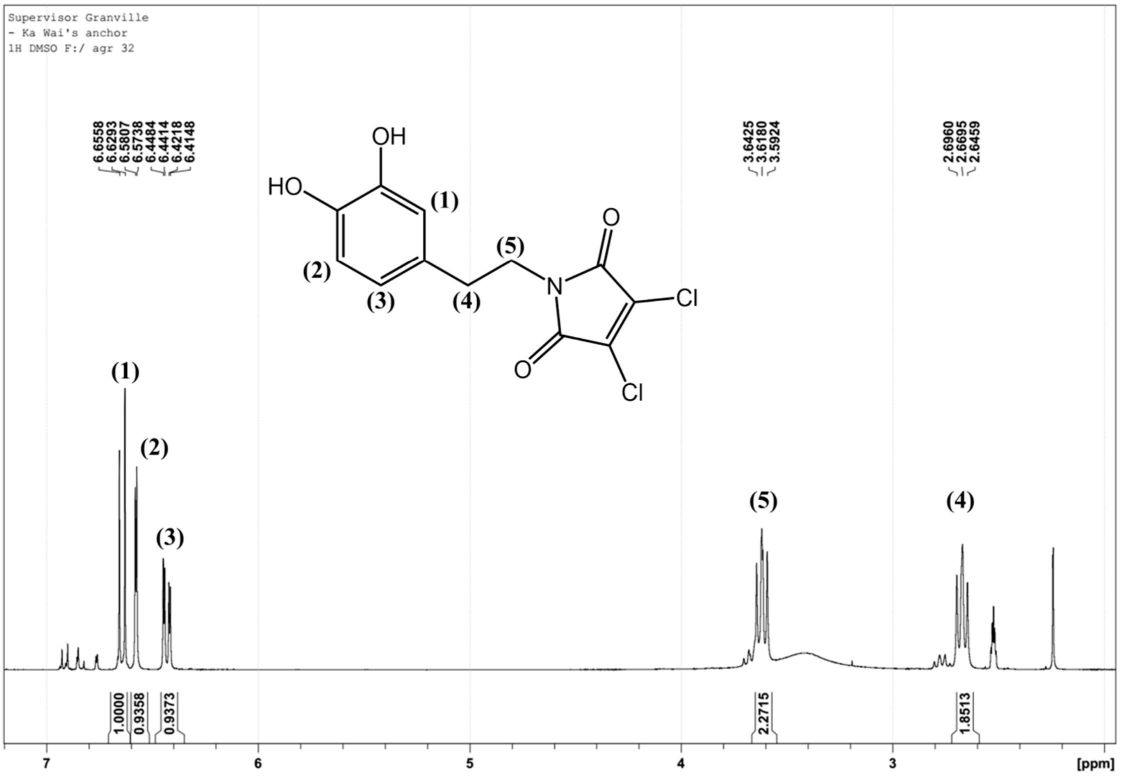

Figure S2. ^{1}H NMR spectrum of the DA-DCMA anchor (in deuterated DMSO).

Table S2. Summary of ^{1}H NMR results of the DA-DCMA anchor.

Peak (δ ppm)	Multiplicity	Structure	Proton integration
6.63			1
6.57	Multiplet	CH (Aromatic)	1
6.43			1
3.62	Triplet	CH_2	2
2.67	Triplet	CH_2	2

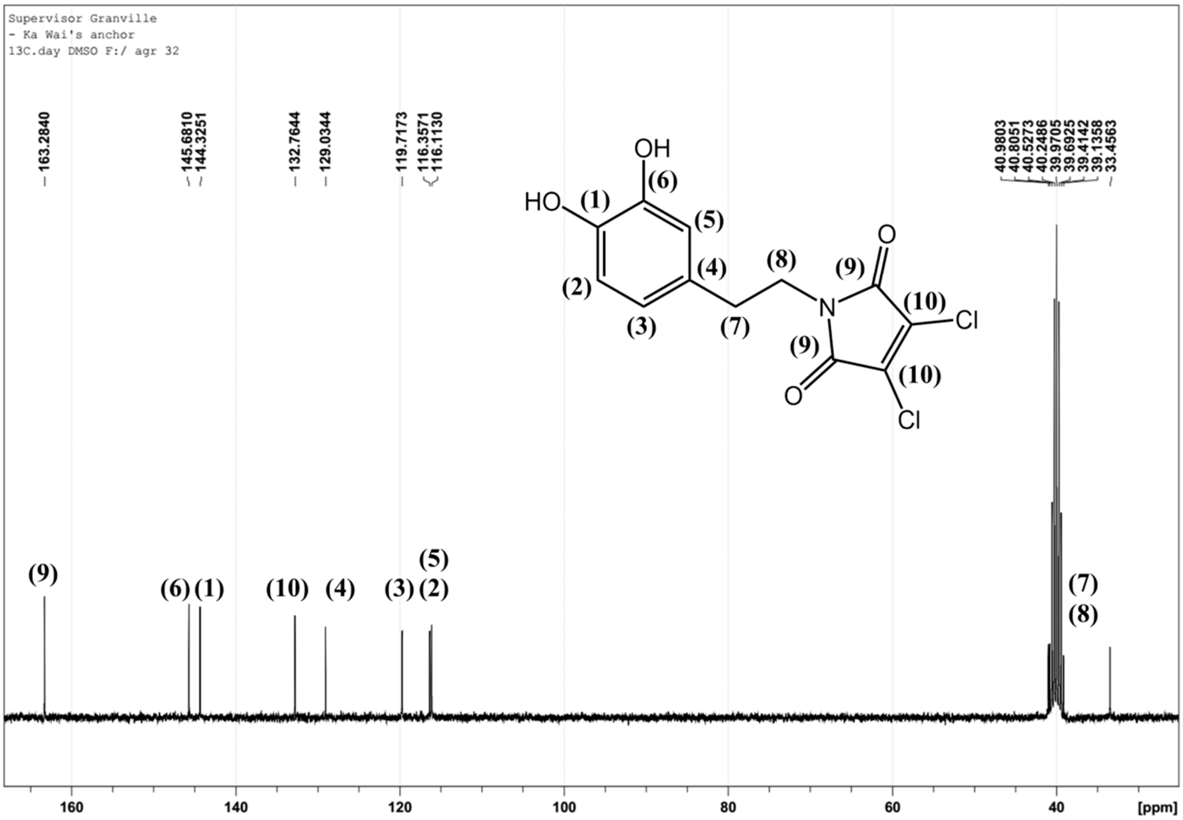

Figure S3. ^{13}C NMR spectrum of the DA-DCMA anchor (in deuterated DMSO).

Table S3. Summary of ^{13}C NMR results of the DA-DCMA anchor.

Peak (δ ppm)	Structure	Carbon integration
163.28	N–C=O	2
145.68	C–OH (Aromatic)	1
144.32	C–OH (Aromatic)	1
132.76	=C–Cl	2
129.03	C (Aromatic)	1
119.72	CH (Aromatic)	1
116.35	CH (Aromatic)	1
116.11	CH (Aromatic)	1

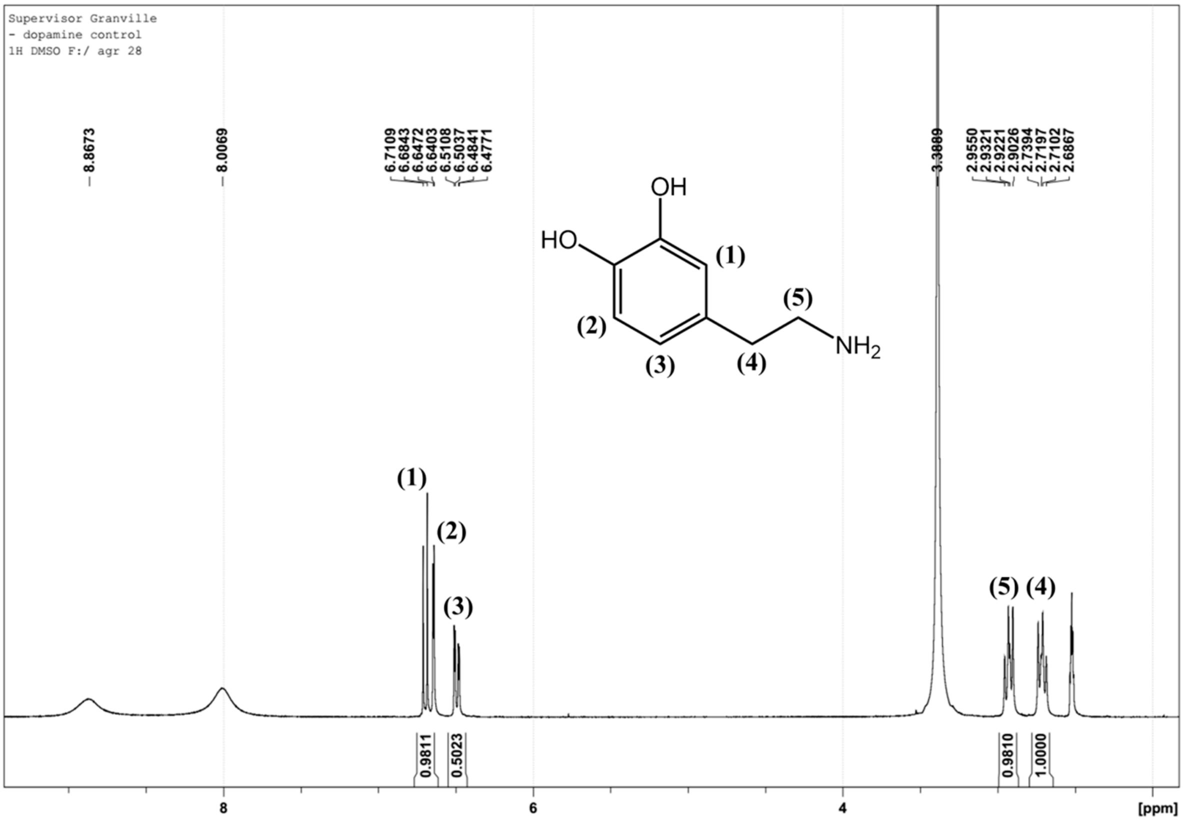

Figure S4. ^{1}H NMR spectrum of dopamine (in deuterated DMSO).

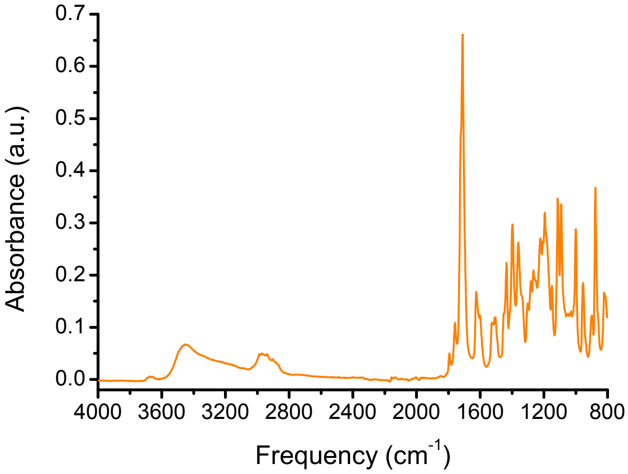

Figure S5. ATR-FTIR spectrum of the DA-DCMA anchor.

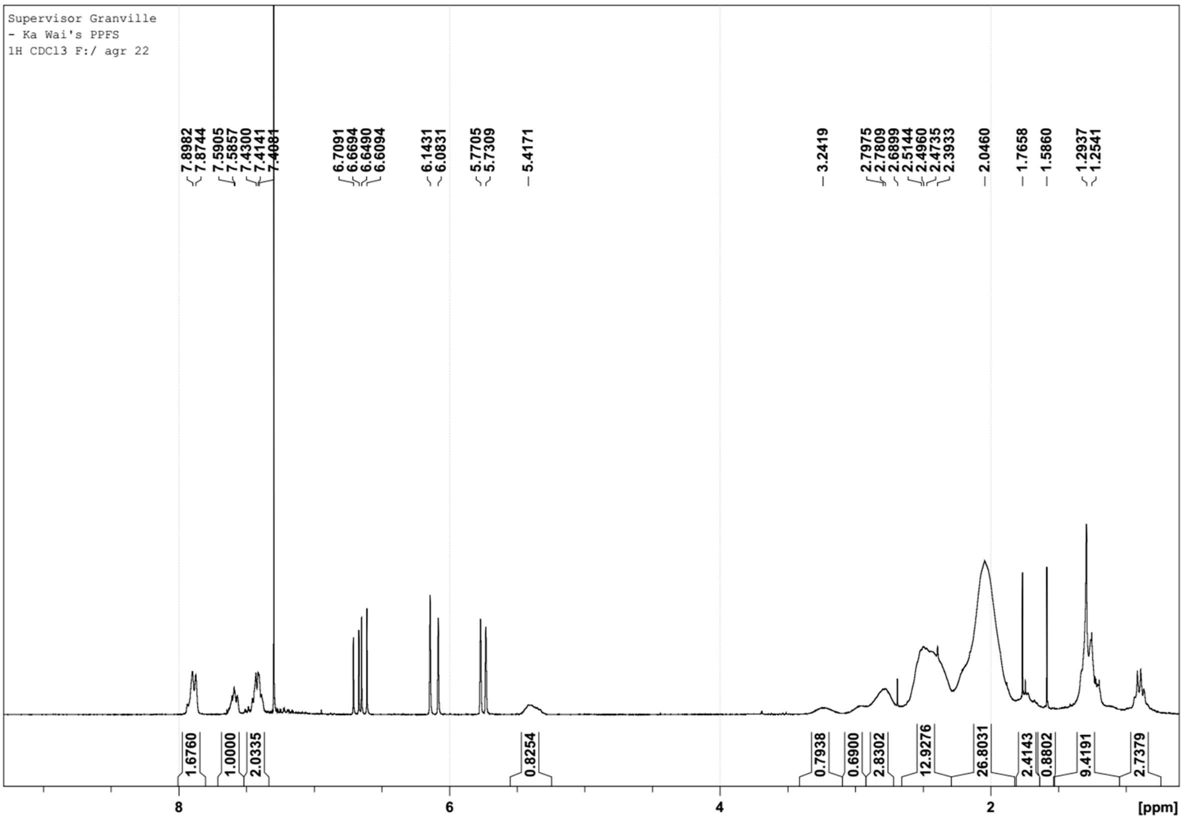

Figure S6. ^{1}H NMR spectrum of the synthesized PPFS (in deuterated chloroform).

Table S4. Summary of 1H NMR results of PPFS.

Peak (δ ppm)	Multiplicity	Structure	Proton integration
7.88			2
7.59	Multiplet	CH (Aromatic-CPADB)	1
7.71			2
2.50	(Broad signal)	CH (Backbone)	13
2.05	(Broad signal)	CH2 (Backbone)	27

Table S5. GPC results of PPFS samples.

Sample status	PPFS (Supplied batch)	
	M_w (g·mol^{-1})	*Đ*
Non-modified	4,394	1.13569
Reduced	7,547	1.15345
Coupled	7,891	1.22284

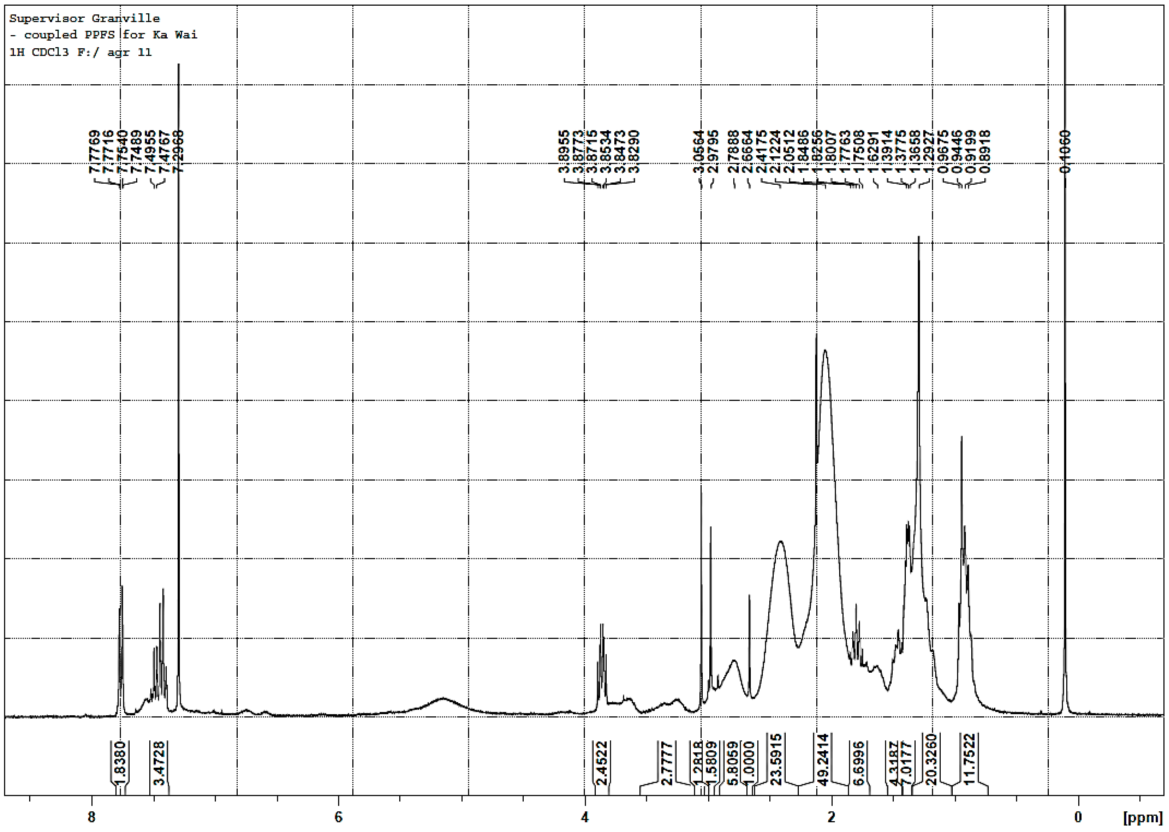

Figure S7. ^1H NMR spectrum of the coupled PPFS (in deuterated chloroform).

Table S5. Peak summary of ^1H NMR results of the coupled PPFS.

Peak (δ ppm)	Multiplicity	Structure	Proton integration
2.42	(Broad signal)	CH (Backbone)	24
2.05	(Broad signal)	CH2 (Backbone)	49

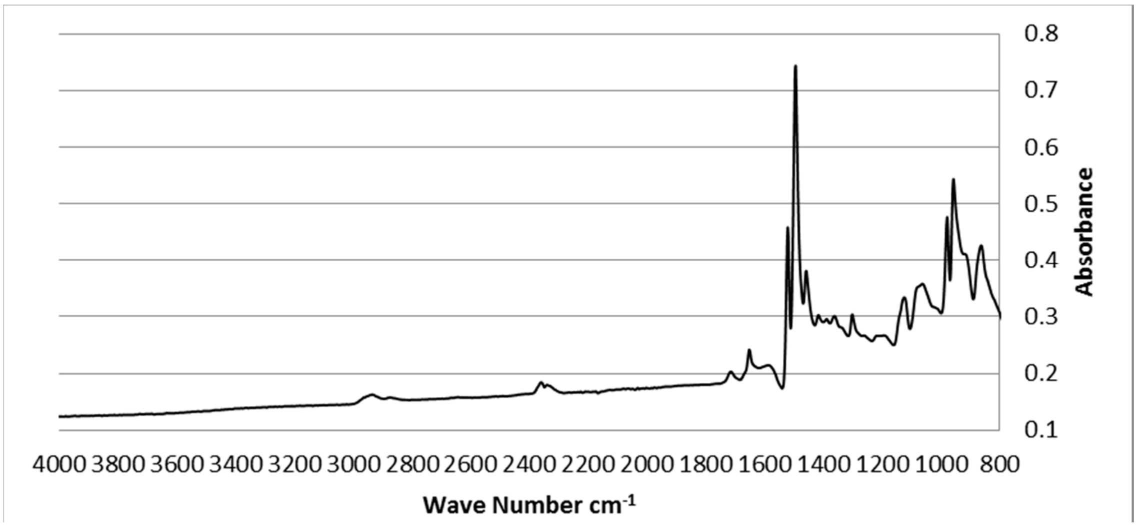

Figure S8. ATR-FTIR spectrum of the purified PPFS-AgNPs.

Table S7. DLS measurement of the prepared PPFS-AgNPs.

Sample name	Dispersant	Z-Average size (d. nm)	PDI	Peak (d. nm)	% Volume
PPFS-AgNPs 1st Batch (From 2nd Batch of MCR AgNPs)	Water	932	0.494	1,179	87.7%
				4,910	10.3%
				182	2.0%
PPFS-AgNPs 1st Batch (From 2nd Batch of MCR AgNPs)	THF	168	0.581	297	32.3%
				80	43.6%
				1,451	21.1%
PPFS-AgNPs 2nd Batch (From 3rd Batch of MCR AgNPs)	THF	121	0.356	21	66.4%
				133	30.7%
				1,651	1.2%

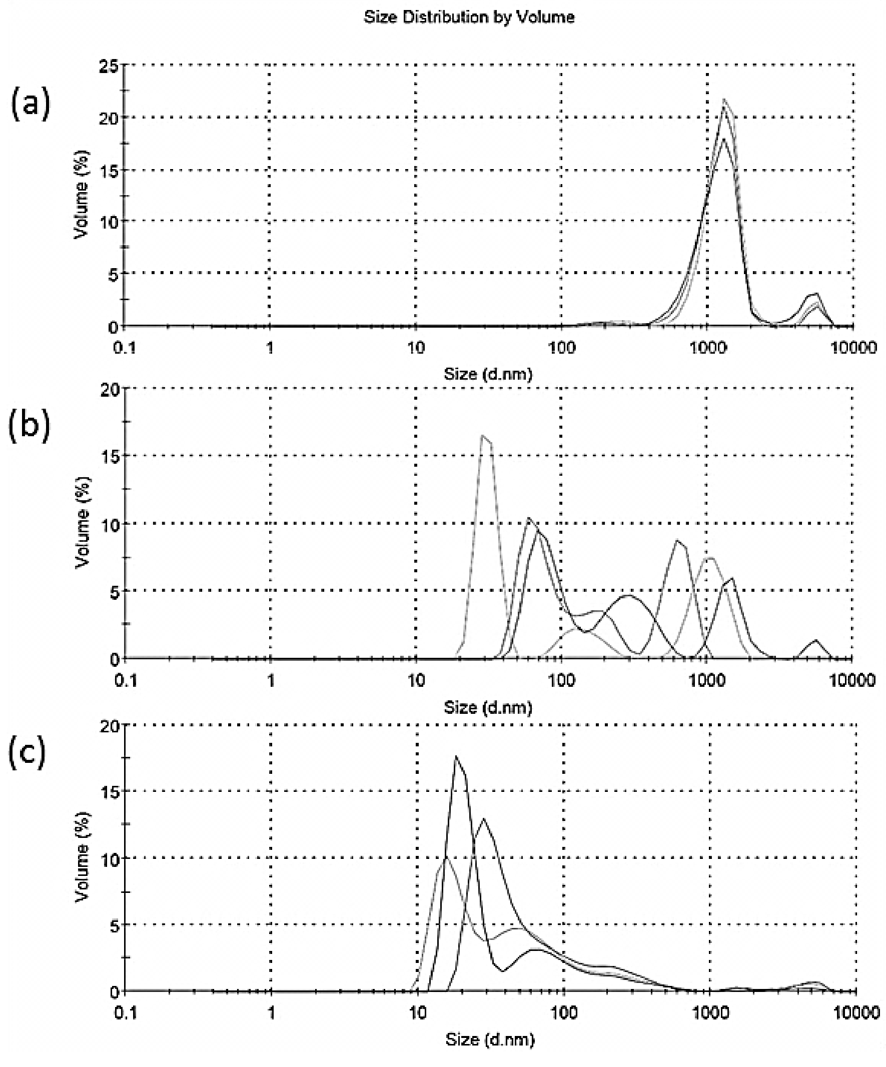

Figure S9. Particle size distribution of PPFS-AgNPs in different dispersants: (**a**) PPFS-AgNPs 1st Batch (in water); (**b**) PPFS-AgNPs 1st Batch (in THF); (**c**) PPFS-AgNPs 2nd Batch (in THF).

Table S6. Determination of the grafting density of the coupled PPFS on AgNPs.

Terms for determining grafting density		**PPFS-AgNPs 2nd Batch**
Sample Mass (mg)		1.117
Molecular Weight of PPFS (mg·mmol^{-1})		4,510
Diameter of AgNP Core (nm)		49.38
Volume of AgNP Core (nm^3)		63,045
Surface Area of AgNP Core (nm^2)		7,660
Density of Silver (g·cm^{-1})		10.5
Mass of AgNP (mg)		6.62×10^{-13}
% AgNPs in Sample		34.12%
% PPFS in Sample		65.88%
Mass of AgNPs in Sample (mg)		0.3811
Mass of PPFS in Sample (mg)		0.7359
No. of particle in Sample		5.76×10^{11}
Mole. of PPFS chain in Sample (mmol)		1.63×10^{-4}
Avogadro's Number		6.02×10^{23}
No. of PPFS Chains in Sample		9.83×10^{16}
Grafting Density	No. of PPFS Chains per NP	170,671
	No. of PPFS Chains per nm^2	22

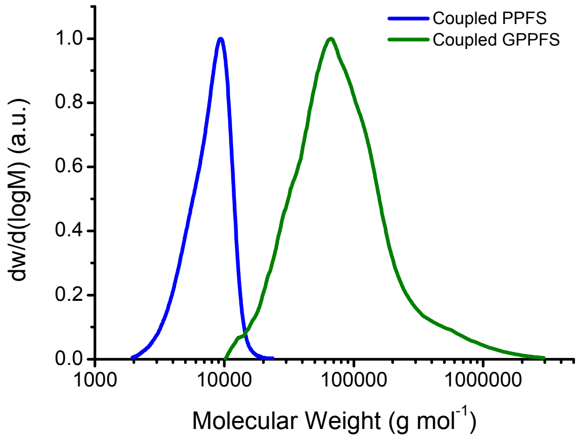

Figure S10. Comparison of molecular weight distribution between GPPFS and coupled PPFS.

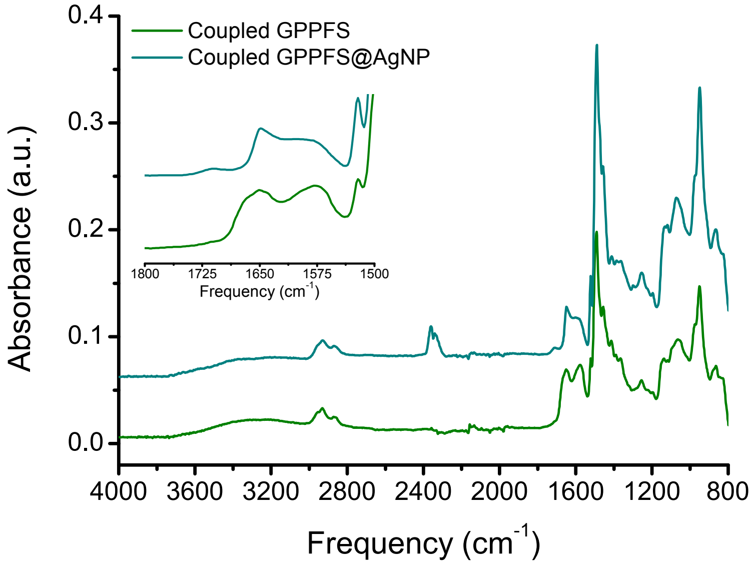

Figure S11. ATR-FTIR spectrum of the isolated product from glycosylation of the coupled PPFS.

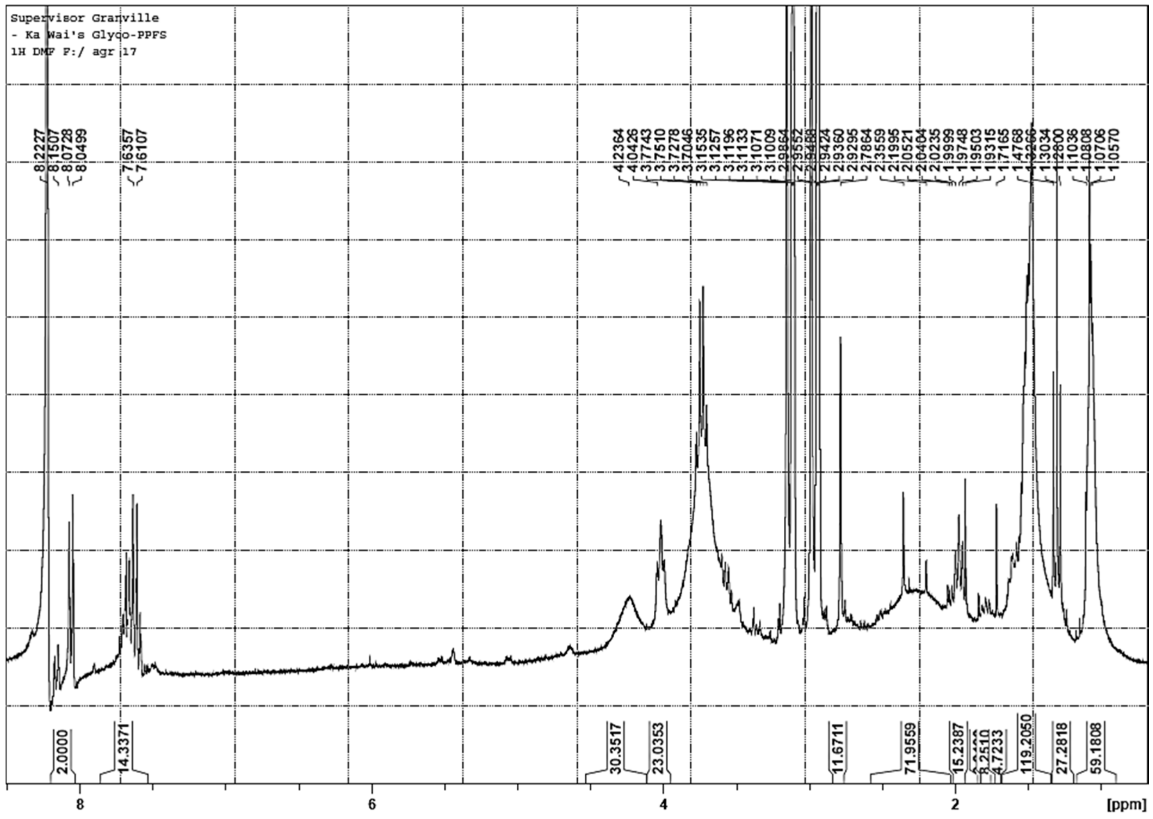

Figure S12. ^{1}H NMR spectrum of the isolated product from glycosylation of the coupled PPFS (in deuterated DMF).

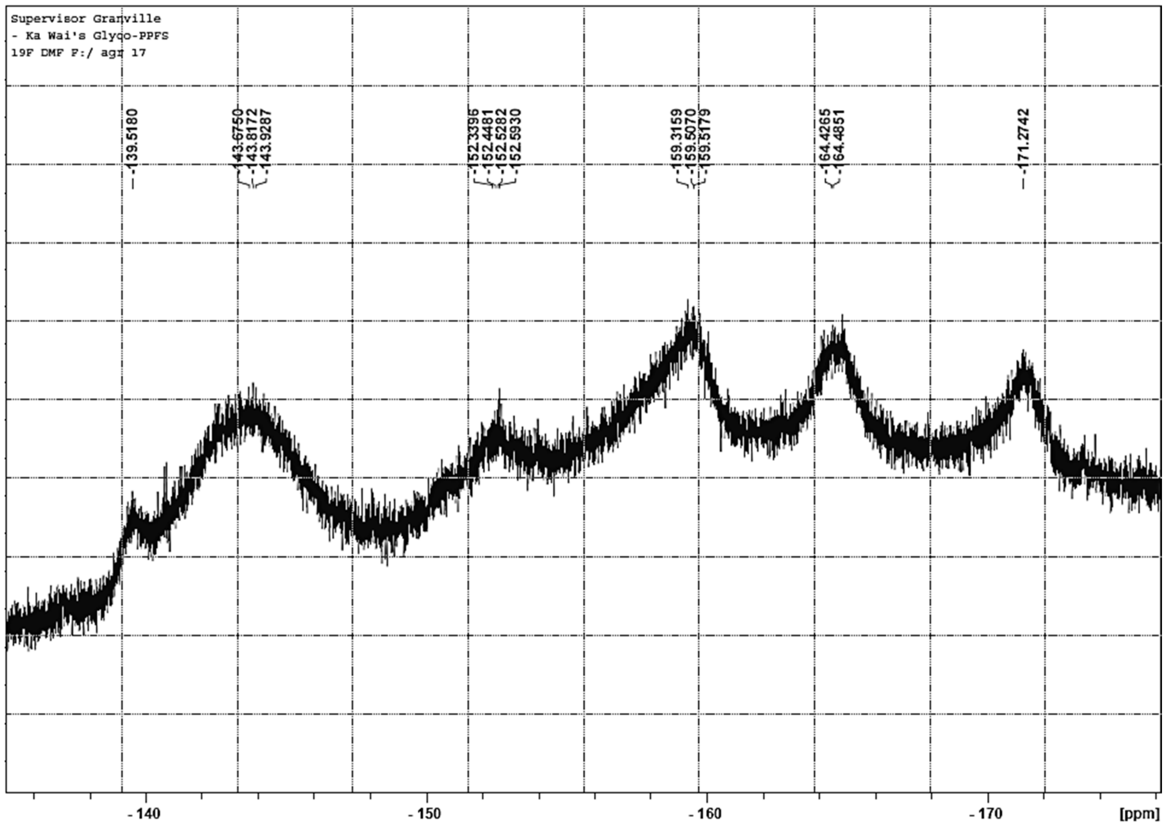

Figure S13. ^{19}F NMR spectrum of the isolated product from glycosylation of the coupled PPFS (in deuterated DMF).

Table S7. Summary of ^{19}F NMR result of the isolated product from glycosylation of the coupled PPFS.

Peak (δ ppm)	Structure	Origin
−139.52	Broad signal (ortho-F)	GPPFS
−143.82	Broad signal (ortho-F)	PPFS
	Broad signal (meta-F)	GPPFS
−159.50	Broad signal (para-F)	PPFS
−164.45	Broad signal (meta-F)	PPFS

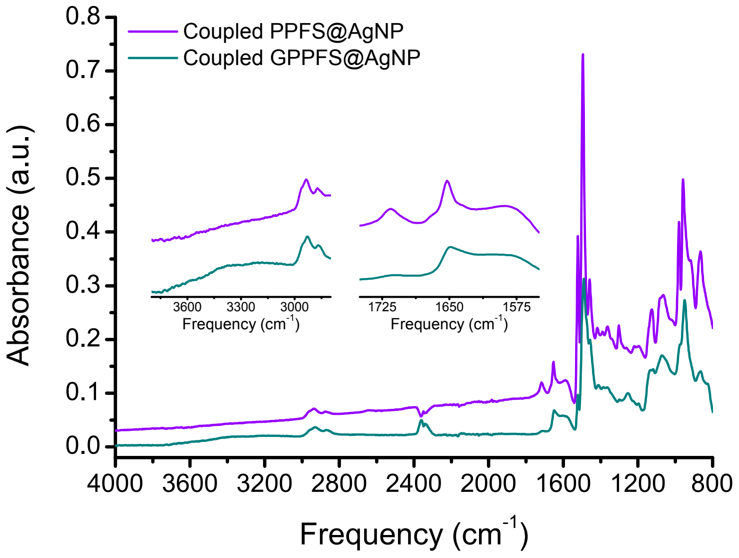

Figure S14. ATR-FTIR spectrum of GPPFS-AgNPs.

© 2016 by the authors; licensee MDPI, Basel, Switzerland. This article is an open access article distributed under the terms and conditions of the Creative Commons by Attribution (CC-BY) license (http://creativecommons.org/licenses/by/4.0/).
